# Potential mobile units drive the horizontal transfer of phytoplasma effector *phyllogen* genes

**DOI:** 10.3389/fgene.2023.1132432

**Published:** 2023-05-11

**Authors:** Ryosuke Tokuda, Nozomu Iwabuchi, Yugo Kitazawa, Takamichi Nijo, Masato Suzuki, Kensaku Maejima, Kenro Oshima, Shigetou Namba, Yasuyuki Yamaji

**Affiliations:** ^1^ Department of Agricultural and Environmental Biology, Graduate School of Agricultural and Life Sciences, The University of Tokyo, Tokyo, Japan; ^2^ Faculty of Bioscience and Applied Chemistry, Hosei University, Tokyo, Japan

**Keywords:** phyllogen, phytoplasma, effector, horizontal gene transfer, potential mobile unit, pathogenicity, symptom

## Abstract

Phytoplasmas are obligate intracellular plant pathogenic bacteria that can induce phyllody, which is a type of abnormal floral organ development. Phytoplasmas possess phyllogens, which are effector proteins that cause phyllody in plants. Phylogenetic comparisons of *phyllogen* and 16S rRNA genes have suggested that *phyllogen* genes undergo horizontal transfer between phytoplasma species and strains. However, the mechanisms and evolutionary implications of this horizontal gene transfer are unclear. Here, we analyzed synteny in *phyllogen* flanking genomic regions from 17 phytoplasma strains that were related to six ‘*Candidatus*’ species, including three strains newly sequenced in this study. Many of the *phyllogens* were flanked by multicopy genes within potential mobile units (PMUs), which are putative transposable elements found in phytoplasmas. The multicopy genes exhibited two distinct patterns of synteny that correlated with the linked *phyllogens*. The low level of sequence identities and partial truncations found among these *phyllogen* flanking genes indicate that the PMU sequences are deteriorating, whereas the highly conserved sequences and functions (e.g., inducing phyllody) of the *phyllogens* suggest that the latter are important for phytoplasma fitness. Furthermore, although their *phyllogens* were similar, PMUs in strains related to ‘*Ca*. P. asteris’ were often located in different regions of the genome. These findings strongly indicate that PMUs drive the horizontal transfer of *phyllogens* among phytoplasma species and strains. These insights improve our understanding of how symptom-determinant genes have been shared among phytoplasmas.

## Introduction

Gene acquisition by horizontal gene transfer (HGT) plays a crucial role in the adaptive evolution of most organisms (Keeling and Palmer, 2008; Soucy et al., 2015). In bacteria, HGT is generally mediated by transposable elements (e.g., phages, plasmids, and transposons) that can travel between host bacteria, as well as by homologous recombination between genomes (Soucy et al., 2015; Drew et al., 2021). Horizontal transfer of single genes or genomic islands can influence bacterial phenotypes and pathogenicity (Melnyk et al., 2019; Gluck-Thaler et al., 2020; Drew et al., 2021). In the genus *Rhizobium*, there is a plasmid carrying genes that can influence root nodulation transfer among different species and determines the host range for root nodule induction (Hooykaas et al., 1982). In the family *Xanthomonadaceae*, colonization of plant vascular tissues prior to systemic infection was associated with horizontal transfer of a single gene, *cbsA* (Gluck-Thaler et al., 2020). The mode of HGT-mediated acquisition of pathogenic genes may have profound implications for pathogenic microbial evolution. These HGTs are mainly inferred using parametric or phylogenetic methods (Ravenhall et al., 2015).

Phytoplasmas [‘*Candidatus* (*Ca.*) Phytoplasma’ spp.] are obligate intracellular plant pathogenic bacteria in the class Mollicutes. Phytoplasmas are transmitted by phloem-feeding insects of the order Hemiptera and can infect more than 1,000 plant species (Marcone, 2014). Although phytoplasma genomes are small and lack many metabolic genes (Oshima et al., 2004), multicopy genes account for 18%–28% of all phytoplasma genes (Oshima et al., 2004; Bai et al., 2006; Kube et al., 2008; Tran-Nguyen et al., 2008). Most of these multicopy genes occur in gene clusters called potential mobile units (PMUs; Bai et al., 2006; Arashida et al., 2008a; Wei et al., 2008). PMUs are often associated with *tra5*, which is a putative transposable gene belonging to the IS3 family, among other genes such as *fliA*, *ssb*, *dam*, *himA*, *hflB*, *smc*, *tmk*, *dnaB*, and *dnaG* (Arashida et al., 2008a). Some PMUs have been reported to transfer in phytoplasma genomes and to exist as probably transposable circular extrachromosomal elements (Arashida et al., 2008a; Toruño et al., 2010; Ku et al., 2013). In addition, PMUs can reportedly undergo HGT between phytoplasma genomes, which mediates the acquisition of novel genes by phytoplasmas (Chung et al., 2013; Ku et al., 2013; Music et al., 2019). Although many PMUs have lost their capacity for HGT due to the deletion of genes such as *tra5*, they are still described as PMUs in many studies, including this one.

Phytoplasma diseases are associated with unique symptoms such as dwarfing, witches’ broom, yellowing, and phyllody (Namba et al., 1993). These symptoms are mainly induced by effector proteins secreted by the phytoplasmas (Hoshi et al., 2009; MacLean et al., 2011; Sugio et al., 2011; Minato et al., 2014; Huang et al., 2021). Phytoplasmas have effectors that cause different disease symptoms. Many of the secreted effector proteins are encoded by PMUs, which phytoplasmas may have acquired by HGT (Sugio and Hogenhout, 2012; Ku et al., 2013). However, to understand the genetics of phytoplasma pathogenicity, it is necessary to confirm that the acquisition of these effectors is associated with HGT of PMUs.

Phyllogens are effector family proteins unique to phytoplasmas that induce phyllody in various eudicots (MacLean et al., 2011; Maejima et al., 2014; Yang et al., 2015; Kitazawa et al., 2017; Iwabuchi et al., 2020). The conserved molecular mechanisms responsible for inducing phyllody have been elucidated (MacLean et al., 2014; Maejima et al., 2014; Kitazawa et al., 2022). Phyllogen genes (*phyllogens*) can be phylogenetically divided into four groups (i.e., phyl-A, -B, -C, and -D) and the evolutionary history of these *phyllogens* differs from that of phytoplasmas, as confirmed by analyses of phytoplasma 16S rRNA gene sequences (Iwabuchi et al., 2020; Kalla et al., 2021). Indeed, different groups of *phyllogens* have been found in the same ‘*Ca*. Phytoplasma’ sp. These findings suggest that *phyllogens* have undergone HGT among various phytoplasma species, thereby enabling these phytoplasmas to induce phyllody (Iwabuchi et al., 2020).

However, gene sequence information is insufficient to establish the evolutionary history of all genes and reveal the mechanism of HGT; therefore, a better understanding of the structure of the gene flanking regions may be necessary (Melnyk et al., 2019; Gluck-Thaler et al., 2020). *Phyllogens* are often found within PMUs (Jomantiene et al., 2007; Sugio and Hogenhout, 2012; Maejima et al., 2014; Wang et al., 2018; Huang et al., 2022); therefore, HGT of *phyllogens* may be mediated by PMUs (Iwabuchi et al., 2020). However, *phyllogen* flanking PMUs have only been analyzed in a few phytoplasma strains (Sugio and Hogenhout, 2012; Wang et al., 2018; Huang et al., 2022), and most *phyllogen* flanking regions have not been studied in detail.

In this study, we analyzed the sequences of *phyllogen* flanking regions from various phytoplasma species and strains, and evaluated similarities to infer the mechanisms and evolutionary history of *phyllogen* acquisition. Sequencing and comparison of *phyllogen* flanking regions revealed synteny among PMU-associated genes for each group of *phyllogens*, indicating that horizontal transfer of PMUs was associated with *phyllogen* acquisition. Furthermore, HGT events were followed by hypermutation, pseudogenesis, and deletion of PMU-associated genes, whereas the sequences of *phyllogens* were highly conserved.

## Materials and methods

### Phytoplasma DNA

DNA samples from ‘*Ca*. P. asteris’ HP, RhY, and PaWB-Japan strains, and from ‘*Ca*. P. phoenicium’ PEY were extracted from infected *Hydrangea* spp. (Takinami et al., 2013), *Rhus javanica* (Takinami et al., 2013), *Paulownia tomentosa* (Kakizawa et al., 2006), and periwinkle (Maejima et al., 2014), respectively.

### Identification of genomic sequences flanking *phyllogens*

The phytoplasma species and strains used in this study are listed in Supplementary Table S1. Contigs of *phyllogen* sequences were created from draft genomes of the HP, RhY, and PaWB-Japan strains sequenced in this study (see Supplementary Methods), as well as from phytoplasma genomic sequences deposited in GenBank (Supplementary Table S1); we performed tBLASTn searches using amino acid sequences from *phyllogen* homologs (Iwabuchi et al., 2020). To determine upstream sequences for the RhY, PaWB-Japan, and PEY strain contigs, we performed polymerase chain reaction (PCR) using primer pairs that targeted each contig and conserved regions of phytoplasma *fliA*, which is frequently found in the first or second open reading frame (ORF) of PMUs (Arashida et al., 2008a; Wang et al., 2018; Supplementary Table S2). The flanking regions of the RhY and PaWB-Japan strain contigs were determined by PCR using primer pairs designed to match the corresponding regions of the HP and PaWB-China genomes, respectively (Supplementary Table S2). The downstream region for the PEY strain was determined by PCR using primer pairs that targeted the *phyllogen* sequence and *dnaG* sequence in the AY-WB strain PMU (Supplementary Table S2). PCR was performed using LA Taq (TaKaRa Bio, Inc., Shiga, Japan) and 0.3 µM of each primer, in accordance with the manufacturer’s instructions. Amplified fragments (>1.5 kbp) were purified and sequenced using PCR primers, followed by primer walking. Amplified fragments (<1.5 kbp) were purified, cloned into a pCR2.1-TOPO vector (Invitrogen, Carlsbad, CA, United States), and sequenced by Sanger sequencing. DNA samples from phytoplasma strains containing phyl-A group *phyllogens* (Supplementary Table S1) were subjected to PCR amplification of the region between *hflB*-like genes with similarity to *hflB* and a region downstream of *phyllogens* using primers described previously (Jomantiene et al., 2007; Supplementary Table S2). The *phyllogen* flanking sequences were added to the draft genomes and deposited in DDBJ under the accession numbers listed in Supplementary Table S1.

### Comparative analysis of *phyllogen* flanking regions

The genomic regions that flanked the *phyllogens* were annotated for protein coding genes using MetaGeneAnnotator (Noguchi et al., 2008) in DFAST (Tanizawa et al., 2018) and analyzed using BLASTp searches restricted to sequences >8,000 bp (Altschul et al., 1990; Camacho et al., 2009). Homologous genes were identified when the E-value, identity, and query-cover between two genes were < 1e−6, >30%, and >75%, respectively. To identify genomic locations of *phyllogen* flanking regions in strains related to ‘*Ca*. P. asteris,’ genomic regions were subjected to comparative analyses with other genomes of strains related to ‘*Ca*. P. asteris’ using BLASTp. The results of these comparative analyses were displayed using Clinker ver. 0.0.21 software to enable gene cluster comparisons (Gilchrist and Chooi., 2021). Pairwise sequence identities were calculated using the Sequence Demarcation Tool (SDT; ver. 1.2) (Muhire et al., 2014). Pseudogenes split into multiple ORFs due to premature stop codons were identified by combining the ORFs.

### Alignment and phylogenetic analyses

The nucleotide sequences of *phyllogen*, *fliA*, *himA*, and *hflB* were picked up from the list in Supplementary Table S3 and were aligned using the MUSCLE algorithm in MEGA X software (Kumar et al., 2018). The neighbor-joining (NJ) method (Saitou and Nei, 1987) was used to create phylogenetic trees of these genes using MEGA X software (Kumar et al., 2018). The whole/draft genome-based phylogenetic analysis of phytoplasma strains related to ‘*Ca*. P. asteris’, except for NJAY strain (GenBank accession number MAPF00000000; completeness of only 91%) and TW strain (GenBank accession number QGKT00000000; draft genome reportedly contaminated with two different strains; Cho et al., 2020), was performed as follows. Single-copy genes shared by the ‘*Ca*. P. asteris’-related phytoplasma genomes (Supplementary Table S3) were identified using SonicParanoid software ver. 1.3.5 (Cosentino and Iwasaki, 2019). The identified homologs were aligned as described above. Aligned sequences were combined for each strain in the same order, and were subjected to phylogenetic analyses using the NJ method as described above.

### *In planta* expression of phyllogen

A modified tobacco rattle virus (TRV)-based gene expression vector system (Iwabuchi et al., 2019) was used to express phyllogen *in planta*. In brief, the PHYL_RP166_ and PHYL_NCHU2019_ sequences were optimized for plant codons and synthesized by ThermoFisher Scientific (Waltham, MA, United States; Supplementary Table S4). The fragments were cloned into pTRV2-2A-sGFP with the primers shown in Supplementary Table S2 by replacing the sGFP region using the NEBuilder HiFi DNA Assembly Cloning kit (New England Biolabs, Ipswich, MA, United States). PHYL_HP_ and PHYL_OY_^E33^ were cloned with the primers shown in Supplementary Table S2 by adding a single amino acid mutation into pTRV2-cloned PHYL_JWB_ (Iwabuchi et al., 2020) and PHYL_OY_ (Iwabuchi et al., 2019), respectively, using the GeneArt site-directed mutagenesis system (Invitrogen). PHYL_PEY_ was cloned with the primers shown in Supplementary Table S2 by incorporating a two-amino acid mutation into pTRV2-cloned PHYL_RP166,_ amplifying the insert by PCR, and amplifying the rest of the plasmid by inverse PCR. The amplified fragments were ligated using the NEBuilder HiFi DNA Assembly Cloning kit (New England Biolabs). *Arabidopsis thaliana* was maintained as described previously (Iwabuchi et al., 2020). *Agrobacterium tumefaciens* EHA105 cells containing pTRV1 and pTRV2-empty, pTRV2-PHYL_OY_, pTRV2-PHYL_RP166_, pTRV2-PHYL_PEY_, pTRV2-PHYL_NCHU2019_, pTRV2-PHYL_HP_, pTRV2-PHYL_JWB,_ or pTRV2-PHYL_OY_^E33^ were adjusted to an OD_600_ of 0.1, mixed at a ratio of 1:1, and co-infiltrated into 2- and 3-week-old *A*. *thaliana* as described previously (Takahashi et al., 2006).

## Results

### The phylogeny of *phyllogens* was consistent with the synteny of the PMUs

**FIGURE 1:**
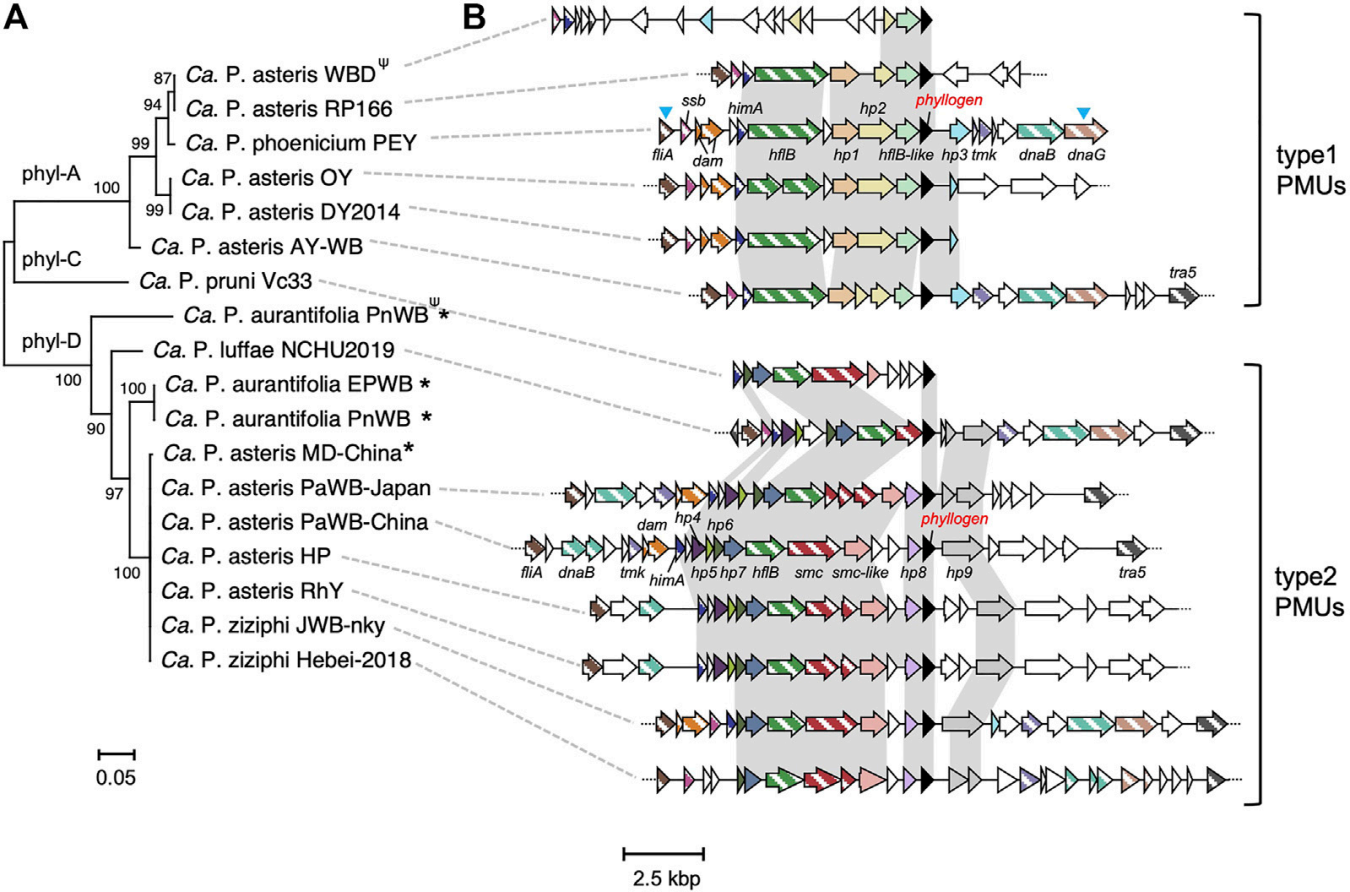
Comparative analysis of *phyllogen* flanking regions. **(A)** Neighbor-joining phylogenetic tree for *phyllogens* with known flanking regions of >8,000 bp, analyzed with the complete deletion option. The MUSCLE multiple alignment algorithm (Kumar et al., 2018) was used to align the *phyllogen* nucleotide sequences. Numbers at the nodes represent bootstrap values for 1,000 replicates (only values >70% are shown). Phylogenetic groups (Iwabuchi et al., 2020) are indicated on the tree branches. The scale bar indicates the number of nucleotide substitutions per site. For *phyllogens* truncated at the C-terminus due to premature stop codons (indicated by *Ψ*), the nucleotide regions after the stop codons were also included in the MUSCLE alignment (Kumar et al., 2018). Phytoplasmas marked with asterisks had *phyllogens* that were not located within PMUs. The ‘*Candidatus* P. ziziphi’ JWB-nky and ‘*Ca*. P. luffae’ NCHU2019 strains had several *phyllogens* with identical sequences; one gene is shown. Full strain names and GenBank accession numbers are listed in Supplementary Table S1. **(B)** Genomic structure of the *phyllogen* flanking regions. Homologous genes present in at least five strains are shown in the same color. *Phyllogens* are shown in black. Diagonal stripe patterns indicate multicopy genes associated with PMUs (Arashida et al., 2008a). The different types of PMU described in this study are indicated on the right. Syntenies unique to the type 1 or type 2 PMUs are highlighted against a light gray background. Open reading frames (ORFs) marked with blue arrowheads have not been completely sequenced. Broken lines at the ends of genomic structures indicate that these regions have been sequenced but are not shown in the figure. The gene names correspond to locus tags in draft genomes (Supplementary Table S8).

To understand the mechanisms involved in the HGT of *phyllogens* across phytoplasmas, we compared *phyllogen* flanking regions among the genomes of 17 phytoplasma strains related to six species (Figure 1; Supplementary Table S1). Strains harboring the phyl-B group *phyllogens* were not analyzed because the corresponding genomes have not been characterized. Prior to this analysis, we generated draft genome data for the ‘*Ca*. P. asteris’ HP, RhY, and PaWB-Japan strains (Supplementary Figure S1; Supplementary Table S5). The draft genome data were used to generate full-length *phyllogen* sequences for the RhY and PaWB-Japan strains, as well as sequences for *phyllogen* flanking regions from the HP, RhY, and PaWB-Japan strains. The detailed results about genome sequencing were described in Supplementary Results. The *phyllogen* flanking regions for the ‘*Ca*. P. phoenicium’ PEY strains were also determined using PCR. Phylogenetic analyses of *phyllogens* showed that the genes of the 17 strains could be separated into three clades (Figure 1A) corresponding to the phyl-A (6 strains), phyl-C (1 strains), and phyl-D groups (10 strains) described by a previous study (Iwabuchi et al., 2020); the RhY and PaWB-Japan *phyllogens* belong to the phyl-D group. In most *phyllogen* flanking regions (all phyl-A and phyl-C, and seven phyl-D group *phyllogens*), there were several PMU-associated genes (*fliA*, *ssb*, *dam*, *himA*, *hflB*, *smc*, *tmk*, *dnaB*, *dnaG*, and *tra5*; Figure 1B; Arashida et al., 2008a). This observation is consistent with previous reports of phytoplasma strains with *phyllogens* within PMUs (Sugio and Hogenhout, 2012; Maejima et al., 2014).

Except for those from the WBD strain, *phyllogen* flanking PMUs frequently contained genes (e.g., *fliA*, *himA*, and *hflB*) in their upstream regions. These PMUs could be categorized as type 1 or 2 based on some characteristic genes and synteny (Figure 1B). PMU typing was already conducted based on the order of *tmk* and *dnaB* in a previous paper (Huang et al., 2022)*.* Since these genes were not all located in the *phyllogen* flanking PMUs, we classified them based on other genes as described below.

The type 1 PMUs were found in six strains (‘*Ca*. P. asteris’ AY-WB, DY 2014, OY, RP166, WBD, and ‘*Ca*. P. phoenicium’ PEY), all of which contained phyl-A group *phyllogens*. These type 1 PMUs also contained *hypothetical protein 1* (*hp1*), *hp2*, and an *hflB*-like gene in their upstream regions (Figure 1B). Additionally, the type 1 PMUs exhibited conserved synteny of *fliA*, *ssb*, *himA*, *hflB*, *hp1*, *hp2*, *hflB*-like, *phyllogen*, and *hp3*, except for the PMUs of the WBD strain. In the WBD strain, only the synteny of *hp2*, *hflB*-like, and the *phyllogens* was conserved. In the ‘*Ca*. P. pruni’ CP and ‘*Ca*. P. trifolii’ CPS strains, as well as in some strains related to ‘*Ca*. P. asteris,’ only the region between the *hflB*-like genes and the intergenic regions downstream of the *phyllogens* were characterized; these regions were conserved in all of these strains, and in the OY and WBD strains (Supplementary Figure S2). This result suggests that the type 1 PMUs that harbor phyl-A group *phyllogens* are conserved in ‘*Ca*. P. pruni,’ ‘*Ca*. P. trifolii,’ ‘*Ca*. P. asteris,’ and ‘*Ca*. P. phoenicium’.

Type 2 PMUs were found in seven strains that have phyl-D group *phyllogens* (‘*Ca*. P. asteris’ HP, RhY, PaWB-Japan, PaWB-China, ‘*Ca*. P. luffae’ NCHU2019, ‘*Ca*. P. ziziphi’ JWB-nky, and Hebei-2018). Type 2 PMUs were also found in the ‘*Ca*. P. pruni’ Vc33 strain, which has a phyl-C group *phyllogen*. The type 2 PMUs contained *hp4*, *hp5*, *hp6*, *hp7, smc*, and *smc*-like genes with similarity to *smc* and *hp8* genes in the upstream region and *hp9* genes in the downstream region. In the PaWB-Japan, PaWB-China, HP, and RhY strains, the type 2 PMUs exhibited conserved synteny of *fliA*, *himA*, *hp4*, *hp5*, *hp6*, *hp7*, *hflB*, *smc*, *smc*-like genes, *hp8*, *phyllogen*, and *hp9*. The JWB-nky and Hebei-2018 strains had another *phyllogen*, and its flanking region also contained genes that were characteristic of type 2 PMUs, although synteny was only partly conserved (Supplementary Figure S3A). The NCHU2019 strain had three type 2 PMUs with the same gene synteny (Supplementary Figure S3B). These results suggest that type 2 PMUs harboring phyl-D or phyl-C group *phyllogens* are conserved in four different species (‘*Ca*. P. asteris,’ ‘*Ca*. P. luffae,’ ‘*Ca*. P. ziziphi,’ and ‘*Ca*. P. pruni’). Additionally, phylogenetic trees based on genes shared by type 1 and 2 PMUs (i.e., *fliA*, *himA*, and *hflB*) indicate that the evolutionary history of these genes differs from that of phytoplasmas. In particular, the *hflB* tree formed two clades, with type 1 and 2 PMU strains (Supplementary Figure S4). Thus, our data indicate that the phylogeny of *phyllogens* is consistent with PMU type (Figure 1).

On the other hand, several *phyllogens* were not located within PMUs (indicated by asterisks; Figure 1). No PMU-associated genes were found in the 20 kbp upstream or downstream of the phyl-D group *phyllogen* in ‘*Ca*. P. aurantifolia’ PnWB, or in the ‘*Ca*. P. asteris’ MD-China strains (except for a *dam* gene in the MD-China strain; Supplementary Figure S6). Additionally, the phyl-D group *phyllogen* in the ‘*Ca*. P. aurantifolia’ EPWB strain was located near the PMU-associated genes, but outside of PMU regions (Supplementary Figure S5).

### *Phyllogen* genes were highly conserved but *phyllogen* flanking PMU genes were not

**FIGURE 2:**
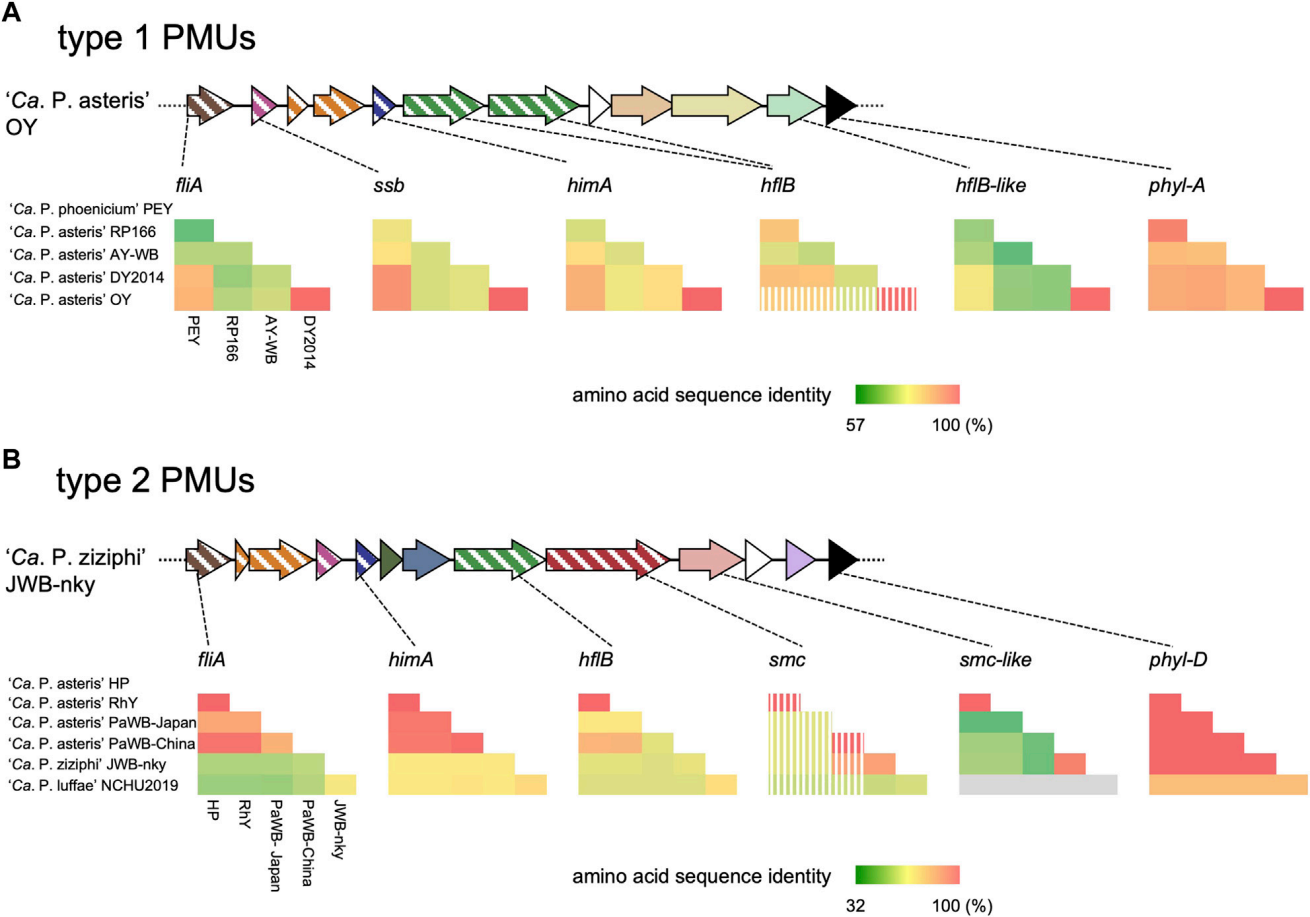
Pairwise sequence comparisons of ORFs flanking phyl-A **(A)** and phyl-D **(B)** group *phyllogens*. Genomic structures are represented as in Figure 1B. Pairwise amino acid sequence identities between phytoplasma species/strains are represented by heatmaps. Strain names are shown on the left and below the heatmaps; full names are listed in Supplementary Table S1. Stripes on the heatmap indicate that truncated genes were used in the analysis. The absence of an ORF from the corresponding genomic region of the NCHU2019 strain is indicated in gray. Results are shown for one of two *phyllogens* for ‘*Ca*. P. ziziphi’ and one of three *phyllogens* for ‘*Ca*. P. luffae.’

Some PMUs contain fewer genes than PMUs reported to exist as probably transposable circular extrachromosomal elements such as AY-WB PMU1 (Toruño et al., 2010) and some PMU-associated genes are truncated (Bai et al., 2006; Ku et al., 2013). To understand the evolution of PMUs harboring *phyllogens*, we analyzed the conservation of genes in type 1 and 2 PMUs using pairwise amino acid sequence comparisons. In the type 1 PMUs, *phyllogens* were highly conserved (87%–100%) between ‘*Ca*. P. asteris’ and ‘*Ca*. P. phoenicium.’ However, genes in the *phyllogen* flanking region (*fliA*, *ssb*, *himA*, *hflB*, *hp1*, *hp2*, and *hflB*-like) exhibited low sequence identities (>57%), except in the closely related OY and DY2014 strains (Supplementary Table S6; Figure 2A). In the type 2 PMUs, *phyllogens* were also highly conserved (91%–100%) among ‘*Ca*. P. asteris’, ‘*Ca*. P. luffae,’ and ‘*Ca*. P. ziziphi,’ except for the Vc33 strain *phyllogen* that belongs to a different phyl-C group. However, the genes in the *phyllogen* flanking regions (*fliA*, *himA*, *hp6*, *hp7*, *hflB*, *smc*, *smc*-like, and *hp9*) exhibited low sequence identities (>32%), except for the closely related HP and RhY strains, and JWB-nky and Hebei-2018 strains (Supplementary Table S7; Figure 2B). Additionally, several PMU-associated genes, including *hflB* in the OY strain and *smc* in the HP, RhY, and PaWB-Japan strains, were truncated by premature stop codons due to frameshifts or single-nucleotide polymorphisms (Figure 1B). Moreover, the RP166, OY, PaWB-Japan, PaWB-China, RhY, and HP strains lacked PMU-associated genes downstream of *phyllogens* (e.g., *tmk*, *dnaB*, *dnaG*, and *tra5*; Figure 1B). These results indicate that although *phyllogens* are highly conserved in both types of PMU, *phyllogen* flanking genes have accumulated mutations, undergone pseudogenesis, and sometimes been lost altogether.

### The phylogenetic relationships among ‘*Ca*. P. asteris’ strains containing *phyllogens* were complex

Next, we focused on relationships among *phyllogens* in ‘*Ca*. P. asteris’ because sequence information and genetic diversity were richer in ‘*Ca*. P. asteris’ than in other phytoplasma species (Iwabuchi et al., 2020). Although many strains related to ‘*Ca*. P. asteris’ had phyl-A group *phyllogens*, several strains had phyl-D group *phyllogens*.

**FIGURE 3:**
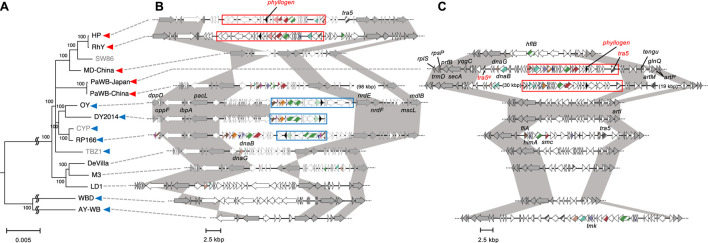
Genomic structure of regions flanking PMUs harboring *phyllogens*. **(A)** Whole/draft genome-based phylogenetic tree of ‘*Ca*. P. asteris’ with the distribution of *phyllogens*. The tree was constructed using the neighbor-joining method and includes single-copy genes shared by all the strains related to ‘*Ca*. P. asteris’ listed in Supplementary Table S3. The ‘*Ca*. P. meliae’ ChTYXIII strain was used as an outgroup. Numbers at the nodes represent bootstrap values for 1,000 replicates (only values >70% are shown). The scale bar indicates the number of nucleotide substitutions per site. The full names and GenBank accession numbers of the strains used in the analysis are listed in Supplementary Table S3. The conserved regions shown in **(B)** and **(C)** were not linked in the strains colored gray. Blue and red arrowheads indicate the presence of phyl-A and phyl-D groups in the genome, respectively. **(B, C)** Genomic structure of the regions surrounding PMUs harboring *phyllogens* from HP, RhY, OY, DY 2014, and RP166 strains **(B)**, and PaWB-Japan and PaWB-China strains **(C)**, compared with other ‘*Ca*. P. asteris’ genomes. ORF structures are represented as in Figure 1B. Dark gray ORFs indicate single-copy ORFs annotated by MetaGeneAnnotator (Noguchi et al., 2008). *Phyllogens* are shown in black. Diagonal stripe patterns indicate multicopy genes associated with PMUs (Arashida et al., 2008a). ORFs that are not associated with PMUs are shown in white. Truncated ORFs are marked by *Ψ*. Conserved regions in strains related to ‘*Ca*. P. asteris’ are shown in dark gray. Type 1 and 2 PMUs that flank *phyllogens* are shown in Figure 1 enclosed by blue and red borders, respectively; the corresponding synteny is highlighted in Figure 1. Broken lines at the ends of genomic structures indicate that these regions have been sequenced but are not shown in the figure.

First, intraspecies evolutionary relationships among strains with *phyllogens* were assessed by analyzing single-copy genes present in all whole/draft genome sequences. In total, 16 strains related to ‘*Ca*. P. asteris’ were analyzed, with a ‘*Ca*. P. meliae’ strain used as an outgroup. A WBD strain, which had recently been proposed for reclassification to ‘*Ca*. P. tritici’ (Zhao et al., 2021), was also used in this analysis. The resulting phylogenetic tree showed that strains related to ‘*Ca*. P. asteris’ formed two clades (Figure 3A). The minor clade comprised the AY-WB and WBD strains, each of which contained phyl-A group *phyllogens*. The major clade comprised four subclades and the TBZ1 strain. Two subclades comprised the strains with phyl-D group *phyllogens*; one of these subclades comprised the HP, RhY, and MD-China strains, and the other comprised the PaWB-Japan and PaWB-China strains. No *phyllogens* were found in the draft genome of the SW86 strain, which belonged to the former subclade. Another subclade comprised the strains containing phyl-A group *phyllogens* (i.e., the CYP, RP166, DY 2014, and OY strains). The final subclade comprised strains with complete (De Villa and M3 strains) or draft (LD1 strain) genomes that lacked *phyllogens*. The TBZ1 strain that contained a phyl-A group *phyllogen* did not belong to any of these subclades. These results indicate that the strains with phyl-D group *phyllogens* formed two subclades in ‘*Ca*. P. asteris’, while the strains with phyl-A group *phyllogens* formed one clade and one subclade. They also indicate that although strains related to ‘*Ca*. P. asteris’ retained the same group of *phyllogens*, at least at the subclade level, the overall relationships were complex.

### PMUs were inserted into different genomic regions in different ‘*Ca*. P. asteris’ subgroups

The positions of the mobile elements within genomes may provide important evolutionary information. Therefore, we analyzed the genomic locations of PMUs in strains related to ‘*Ca*. P. asteris,’ based on their flanking genomic regions. First, we analyzed the genomic locations of the type 1 PMUs. The PMUs and surrounding genomic regions in the OY, DY 2014, RP166, and AY-WB strains are shown in Figure 3B; Supplementary Figure S6B. The type 1 PMUs of the OY, DY 2014, and RP166 strains, which belong to the same subclade as ‘*Ca*. P. asteris’ (Figure 3A), are flanked by several single-copy genes. These genes include *dppD*, *oppF*, *ibpA*, and *pacL* in the upstream region, and *nrdE*, *nrdF*, *mscL*, and *mdlB* in the downstream region (Figure 3B). The syntenies of these single-copy genes were also conserved in the other ‘*Ca*. P. asteris’ genomes, but there were no *phyllogens* in the corresponding regions, except in the HP and RhY strains. However, type 2 PMUs harboring *phyllogens* were found in these corresponding genomic regions. The type 1 PMU in the AY-WB strain was in a different genomic region, and was flanked by several single-copy genes including *pdhC*, *acoL*, *tatD*, and *plsX* in the upstream region and *rpsD*, *mgtA*, *degV*, and *tsaD* in the downstream region (Supplementary Figure S6B). The syntenies of these genes were conserved in other ‘*Ca*. P. asteris’ genomes, although no type 1 PMUs or *phyllogens* were found. These results indicate that the type 1 PMUs were inserted into different regions in the AY-WB and OY/DY2014/RP166 strains. Next, we analyzed the genomic locations of the type 2 PMUs. The PMUs and surrounding genomic regions in the PaWB-Japan and PaWB-China strains, which belong to the same subgroup, are shown in Figure 3C. These PMUs were delimited by a complete and truncated *tra5* gene downstream and upstream of the *phyllogens*, respectively (Bai et al., 2006). Several single-copy genes were also found near the PMUs, but these genes were different from those surrounding the type 1 PMUs; they included *rpsP*, *prfB*, *secA*, and *ypgC* in the upstream region and *tengu*, *glnQ*, and *artl* in the downstream region (Figure 3C). Although the syntenies of these single-copy genes were also conserved in other ‘*Ca*. P. asteris’ genomes, there were no type 2 PMUs or *phyllogens*. The genomic region between the *ypgC* and *tengu* genes in the OY, DY 2014, De Villa, M3, and WBD strains was <13 kbp, which was much shorter than in the PaWB-Japan (35 kbp) and PaWB-China (67 kbp) strains. Several PMU-associated genes were found in this genomic region in the RP166 and AY-WB strains; however, the syntenies of these genes differed from those of the type 2 PMUs (Figure 3C). On the other hand, the type 2 PMUs in the HP and RhY strains were flanked by several single-copy genes different from those present in the PaWB strains (Figure 3B). The phyl-D group *phyllogen* present in the MD-China strain was also flanked by single-copy genes different from the genes flanking other *phyllogens* (Supplementary Figure S6). These results indicate that type 2 PMUs were inserted into different regions in the PaWB-Japan/PaWB-China, MD-China, and HP/RhY strains.

**FIGURE 4:**
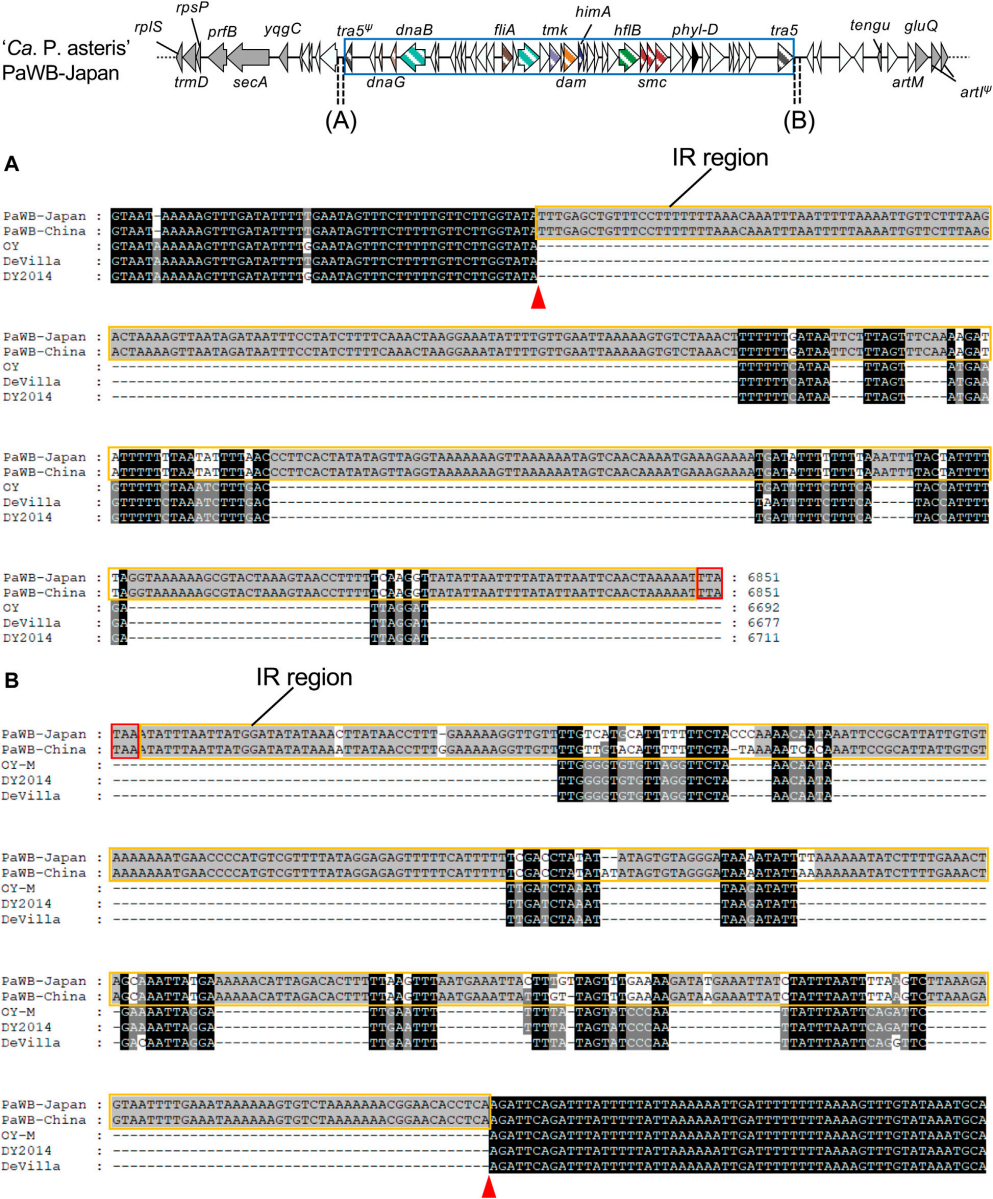
Nucleotide sequence alignment of the downstream sequences, beginning at stop codons of truncated *tra5* in upstream regions **(A)** or intact *tra5* in downstream regions **(B)** of *phyllogens*. Stop codons in upstream regions of truncated *tra5* genes were identified by comparing nucleotide sequences with the intact *tra5* gene. *Tra5* stop codons are enclosed by red borders. Inverted repeat regions are enclosed by orange borders. Putative PMU boundaries are marked with red arrowheads. Genomic structures of the *phyllogen* flanking regions in the PaWB-Japan strain are represented as in Figure 3. PMUs are enclosed by blue borders.

To correlate the inserted type 2 PMUs with other genomes at the nucleotide level, the upstream and downstream sequences of the PMUs in the PaWB strains were aligned with the corresponding genomic regions in the OY, De Villa, and DY2014 strains. The alignments identified regions of approximately 350 bp at both ends of the insertions that contained inverted repeat sequences, which is characteristic of transposon insertion (Szeverényi et al., 2003), and these inverted repeats were conserved between the PaWB strains (Figure 4; Supplementary Figure S7). Although these inverted repeat sequences were not identical in the other three strains, there were nearby sequences with strong similarity among these genomes (Figure 4), suggesting that PMU boundaries were located near the inverted repeats. These results indicate that a type 2 PMU was inserted at this position in the common ancestor of the PaWB strains.

### Functions of PMU-associated phyllogens were conserved

**FIGURE 5:**
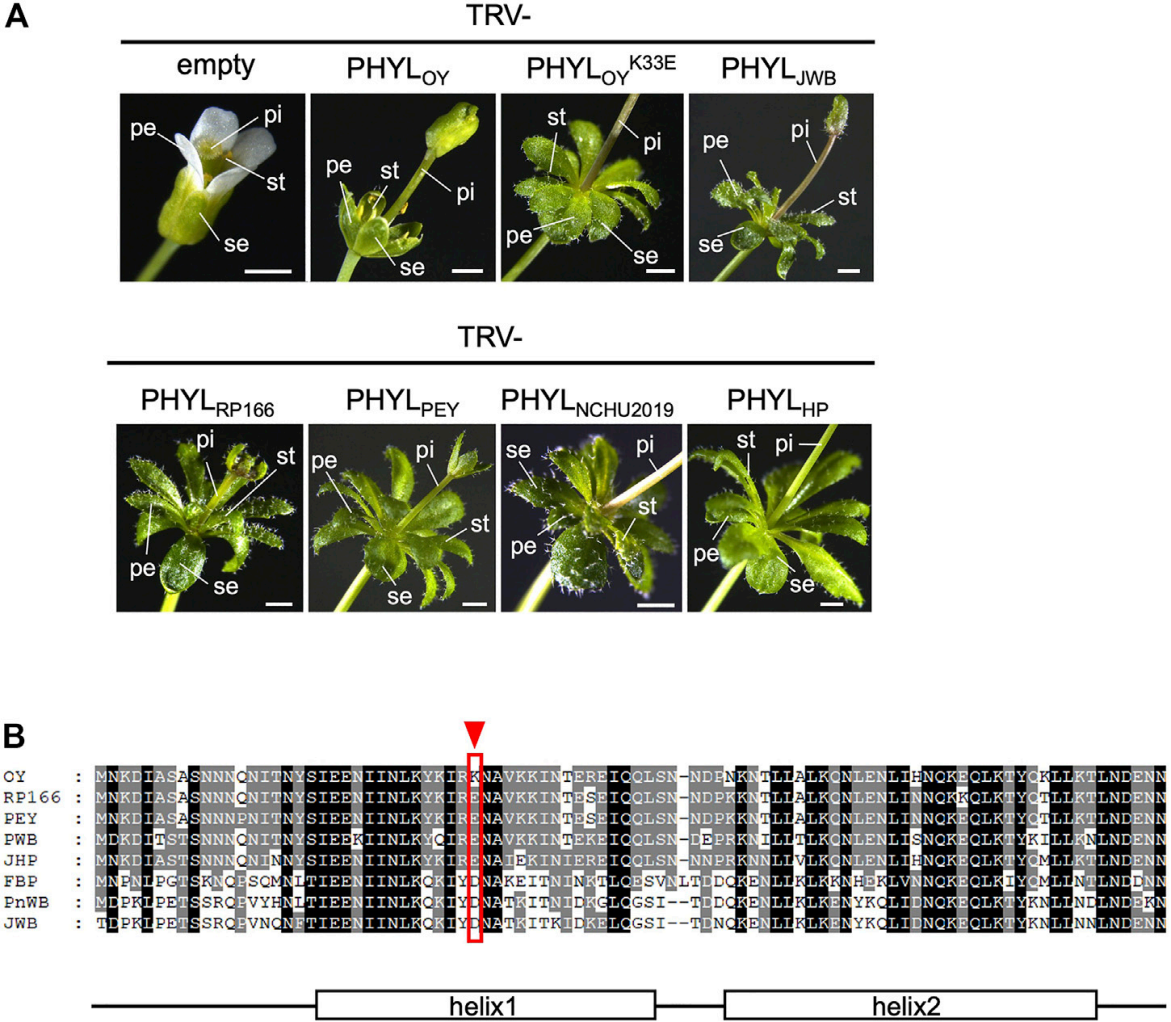
**(A)** Functional analysis of *phyllogens* associated with PMUs and PHYL_OY_^K33E^. *Arabidopsis thaliana* plants were infected with the tobacco rattle virus (TRV) vectors carrying *phyllogens* from either the phyl-A or phyl-D group. The following floral organs are shown: sepals (se), petals (pe), stamens (st), and pistils (pi). White scale bar: 1 mm. **(B)** Amino acid alignment of the secreted regions of PHYL_OY_ and the phyl-A or phyl-D group phyllogens, which induce severe phyllody. The red arrowhead and border indicate a unique polymorphism at position 33 of PHYL_OY_. Consensus secondary structure elements of phyllogens are shown (Iwabuchi et al., 2019).

Comparative analyses revealed conservation of amino acid sequences of the PMU-associated phyllogens. Thus, we examined functional conservation of the phyllogens by testing the capacity of various phyllogens to induce phyllody. We tested the phyl-A group phyllogens of RP166 (PHYL_RP166_) and the PEY strains (PHYL_PEY_), as well as the phyl-D group phyllogens of NCHU2019 (PHYL_NCHU2019_) and the HP strains (PHYL_HP_). We used the PHYL_OY_ phyllogen of the OY strain (phyl-A group) and the PHYL_JWB_ phyllogen of the JWB strain (phyl-D group) as controls known to induce phyllody (Iwabuchi et al., 2020). Each phyllogen was expressed in *A*. *thaliana* using a TRV-based gene expression vector system. All of the tested phyllogens converted sepals, petals, and stamens into leaf-like structures with stellate trichomes, changed pistils into secondary vegetative shoot-like structures, and enlarged flowers (Figure 5A), as did the PHYL_JWB_ phyllogen control. These results showed that the functions of the type 1 and 2 PMU-associated phyllogens were as highly conserved as their sequences.

Interestingly, in the PHYL_OY_ expression test, the stamens of most flowers were not converted into leaf-like structures, the other floral organs had fewer trichomes, and the flowers did not enlarge (Figure 5A). This suggested that PHYL_OY_ exhibited a reduced capacity to induce phyllody. Sequence comparisons revealed that PHYL_OY_ had a unique polymorphism at position 33 (lysine; Figure 5B). A PHYL_OY_ mutant with a reciprocal substitution at this position (glutamate; PHYL_OY_^K33E^) induced phyllody to the same extent as the other homologs (Figure 5A). Therefore, this amino acid is important for strong induction of phyllody.

## Discussion

### Interspecies acquisition of *phyllogens via* horizontal transfer of PMUs

The apparent differences between the evolutionary histories of *phyllogens* and 16S rRNA genes in phytoplasmas suggest that *phyllogens* may have undergone HGT (Iwabuchi et al., 2020; Kalla et al., 2021). However, the mechanisms involved were previously unclear due to the lack of *phyllogen* flanking-region sequence data. In this study, we determined draft genomes for three strains and analyzed *phyllogen* flanking regions from 17 phytoplasma strains related to six different species. We found that most *phyllogens* were associated with PMUs (Figure 1). Furthermore, *phyllogen* groupings correlated closely with the types of PMU (Figure 1). These results indicate that PMUs drive the horizontal transfer of distinct groups of *phyllogens* between phytoplasma species and strains. This study demonstrates that the symptom-determinant effectors of phytoplasmas are evolutionarily correlated with the types of PMU rather than the phytoplasma genomes.

In addition to phyllogens, three other effectors (TENGU, SAP11, and SAP05) have been linked experimentally with phytoplasma virulence (Hoshi et al., 2009; Sugawara et al., 2013; Minato et al., 2014; Cho et al., 2019; Huang et al., 2021). Among these, the SAP11 and SAP05 genes are also located near PMUs; they are conserved in various species (Sugio and Hogenhout., 2012; Cho et al., 2019; Huang et al., 2022) and have phylogenetic trees that differ from those of phytoplasmas (Chang et al., 2018; Huang et al., 2022). These observations suggest that PMU-mediated HGT may play a major role in the transfer of virulence genes across phytoplasma species.

Many studies have reported HGT of bacterial virulence genes based on genomic analyses of individual species (Ma et al., 2006; McCann and Guttman, 2008; Drew et al., 2021), but few have shown how HGT can occur *via* transposable elements (such as transposons) by comparing the genomes of different bacterial species. Thus, this study provides new insight into the mechanisms of HGT of virulence genes.

### *Phyllogen* sequences and functions are conserved but PMU sequences may deteriorate

Genetic elements may become immobilized due to inactivation or deletion of genes necessary for their transfer (Dobrindt et al., 2004). Degeneration of PMUs has also been reported in several phytoplasma strains, including AY-WB (Bai et al., 2006; Ku et al., 2013). In this study, some PMUs harboring *phyllogens* lacked *tra5* (Figure 1B). In addition, some PMU genes had accumulated mutations, were truncated, or had been lost altogether (Figures 1B, 2), including genes with putative roles in DNA recombination, replication, and transposition (Arashida et al., 2008a; Ku et al., 2013). Therefore, most of these PMUs may begin to lose their capacity for transposition after the *phyllogens* have been acquired. However, the type 2 PMUs of the PaWB-Japan and PaWB-China strains retained their *tra5* genes and inverted repeat-like sequences at both ends (Figures 3, 4), suggesting that they had also retained their capacity for transposition.

Despite the deterioration of PMU-associated genes, *phyllogens* from different species had highly conserved sequences, especially *phyllogens* from the phyl-A and -D groups (Figure 2). Furthermore, phyllogen functions were conserved (Figure 5). Several *phyllogens* were not located within PMUs (Figure 1A; Chung et al., 2013; Luo et al., 2022) and their flanking regions exhibited considerable variation (data not shown), suggesting that after *phyllogen* transfer, PMU sequences may deteriorate completely. Therefore, *phyllogens* may become fixed in many different phytoplasma genomes after their acquisition, suggesting that they are crucial for their hosts. Previous studies have shown that phyllody symptoms facilitate phytoplasma accumulation/localization within host plants (Arashida et al., 2008b; Su et al., 2011), and phyllogens can attract insect vectors (MacLean et al., 2014). Although further studies are needed to investigate the roles of phyllody symptoms in host adaptability, the fact that *phyllogens* have been strongly conserved throughout evolution suggests that they are critical for phytoplasma survival.

### Evolutionary history of *phyllogen* acquisition in ‘*Ca*. P. asteris’

To date, three different groups of *phyllogens* have been identified in strains related to ‘*Ca*. P. asteris’ (phyl-A, -B, and -D; Iwabuchi et al., 2020). In this study, we found that the acquisition of these *phyllogens* was a complex process, at least for phyl-A and phyl-D. In ‘*Ca*. P. asteris,’ we identified one subclade comprising strains that retained phyl-A *phyllogens* and two subclades comprising strains that retained phyl-D *phyllogens* (Figure 3A). In the former subclade, the PMUs retaining phyl-A were found in the same region of the genomes (Figure 3), suggesting that these PMUs were acquired by their common ancestor. The same region also surrounded PMUs of the HP and RhY strains (Figure 3). Therefore, this region may be a hot spot for genome rearrangement in phytoplasmas, as described by a previous study (Arashida et al., 2008b). PMUs retaining phyl-D were found in different genomic regions in the PaWB-Japan/PaWB-China, MD-China, and HP/RhY strains (Figure 3; Supplementary Figure S6). This observation suggests that PMUs harboring *phyllogens* either moved frequently within the genome or were acquired separately. The PaWB strains had complete PMUs retaining phyl-D, suggesting that these were acquired more recently than the PMUs in the HP and RhY strains. Therefore, the latter hypothesis seems more plausible. We could not determine the acquisition order of the phyl-A and phyl-D group *phyllogens* in ‘*Ca*. P. asteris’ due to insufficient genomic data. Therefore, further studies of *phyllogen* flanking regions and their genomic positions are needed.

HGT may occur between organisms that share an ecological niche, irrespective of their phylogenetic relationships (Polz et al., 2013). Phylogenetic analyses have shown that all of the phyl-D group *phyllogens* of ‘*Ca*. P. asteris’ are most closely related to *phyllogens* of ‘*Ca*. P. ziziphi’ (Iwabuchi et al., 2020; Figure 1A). Furthermore, several lines of evidence indicate that strains related to ‘*Ca*. P. asteris’ and ‘*Ca*. P. ziziphi’ share common ecological niches. First, the distribution of ‘*Ca*. P. ziziphi’ is limited to eastern and southern Asia (Jung et al., 2003; Rao et al., 2017), as is the distribution of strains related to ‘*Ca*. P. asteris’ that retain phyl-D (Kakizawa et al., 2006; Takinami et al., 2013; Luo et al., 2022). Second, co-infection of ‘*Ca*. P. asteris’ and ‘*Ca*. P. ziziphi’ has been reported in jujube plants (Sun et al., 2013). Third, *Hishimonus sellatus*, a vector of ‘*Ca*. P. ziziphi,’ can transmit two strains related to ‘*Ca*. P. asteris’ that retain phyl-D (RhY and MD-China; Tanaka et al., 2000; Kusunoki et al., 2002). Therefore, type 2 PMUs in ‘*Ca*. P. asteris’ may be acquired from ‘*Ca*. P. ziziphi’ in the same ecological niche. Further accumulation of information on the synteny of PMUs will reveal how PMUs and symptom-determinant genes located in them are shared among phytoplasmas in the same niche.

## Data Availability

The datasets presented in this study can be found in online repositories. The names of the repository/repositories and accession number(s) can be found below: https://www.ddbj.nig.ac.jp/, BSDA00000000; https://www.ddbj.nig.ac.jp/, BSCX00000000; https://www.ddbj.nig.ac.jp/, BSCY00000000; https://www.ddbj.nig.ac.jp/, LC740440; https://www.ddbj.nig.ac.jp/, LC740441; https://www.ddbj.nig.ac.jp/, LC740442; https://www.ddbj.nig.ac.jp/, LC740443; https://www.ddbj.nig.ac.jp/, LC740444; https://www.ddbj.nig.ac.jp/, LC740445; https://www.ddbj.nig.ac.jp/, LC740446; https://www.ddbj.nig.ac.jp/, LC740447; https://www.ddbj.nig.ac.jp/, LC740448; https://www.ddbj.nig.ac.jp/, LC740449; https://www.ddbj.nig.ac.jp/, LC740450; https://www.ddbj.nig.ac.jp/, LC740451; https://www.ddbj.nig.ac.jp/, LC740452; https://www.ddbj.nig.ac.jp/, LC740453.
