# Supplementary material for: Potential mobile units drive the horizontal transfer of phytoplasma effector phyllogen genes
Source: Front Genet. 2023 May 11;14:1132432. doi: 10.3389/fgene.2023.1132432 (PMC10210161; doi:10.3389/fgene.2023.1132432)
Supplement: Supplementary file 1 [file Presentation1.pptx]

## Slide 1
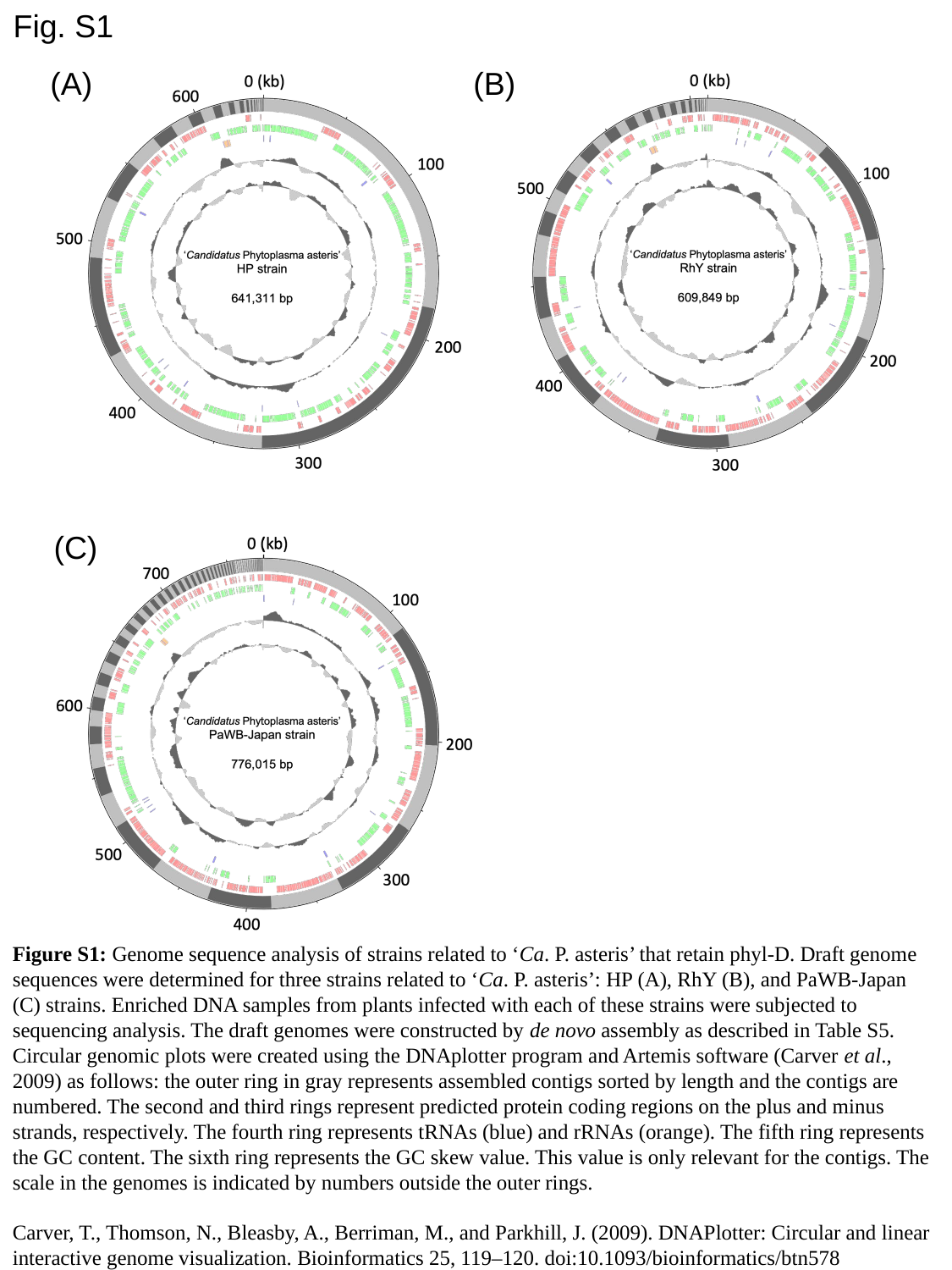

Fig. S1
(A)
(B)
(C)
Figure S1: Genome sequence analysis of strains related to ‘Ca. P. asteris’ that retain phyl-D. Draft genome sequences were determined for three strains related to ‘Ca. P. asteris’: HP (A), RhY (B), and PaWB-Japan (C) strains. Enriched DNA samples from plants infected with each of these strains were subjected to sequencing analysis. The draft genomes were constructed by de novo assembly as described in Table S5. Circular genomic plots were created using the DNAplotter program and Artemis software (Carver et al., 2009) as follows: the outer ring in gray represents assembled contigs sorted by length and the contigs are numbered. The second and third rings represent predicted protein coding regions on the plus and minus strands, respectively. The fourth ring represents tRNAs (blue) and rRNAs (orange). The fifth ring represents the GC content. The sixth ring represents the GC skew value. This value is only relevant for the contigs. The scale in the genomes is indicated by numbers outside the outer rings.
Carver, T., Thomson, N., Bleasby, A., Berriman, M., and Parkhill, J. (2009). DNAPlotter: Circular and linear interactive genome visualization. Bioinformatics 25, 119–120. doi:10.1093/bioinformatics/btn578

## Slide 2
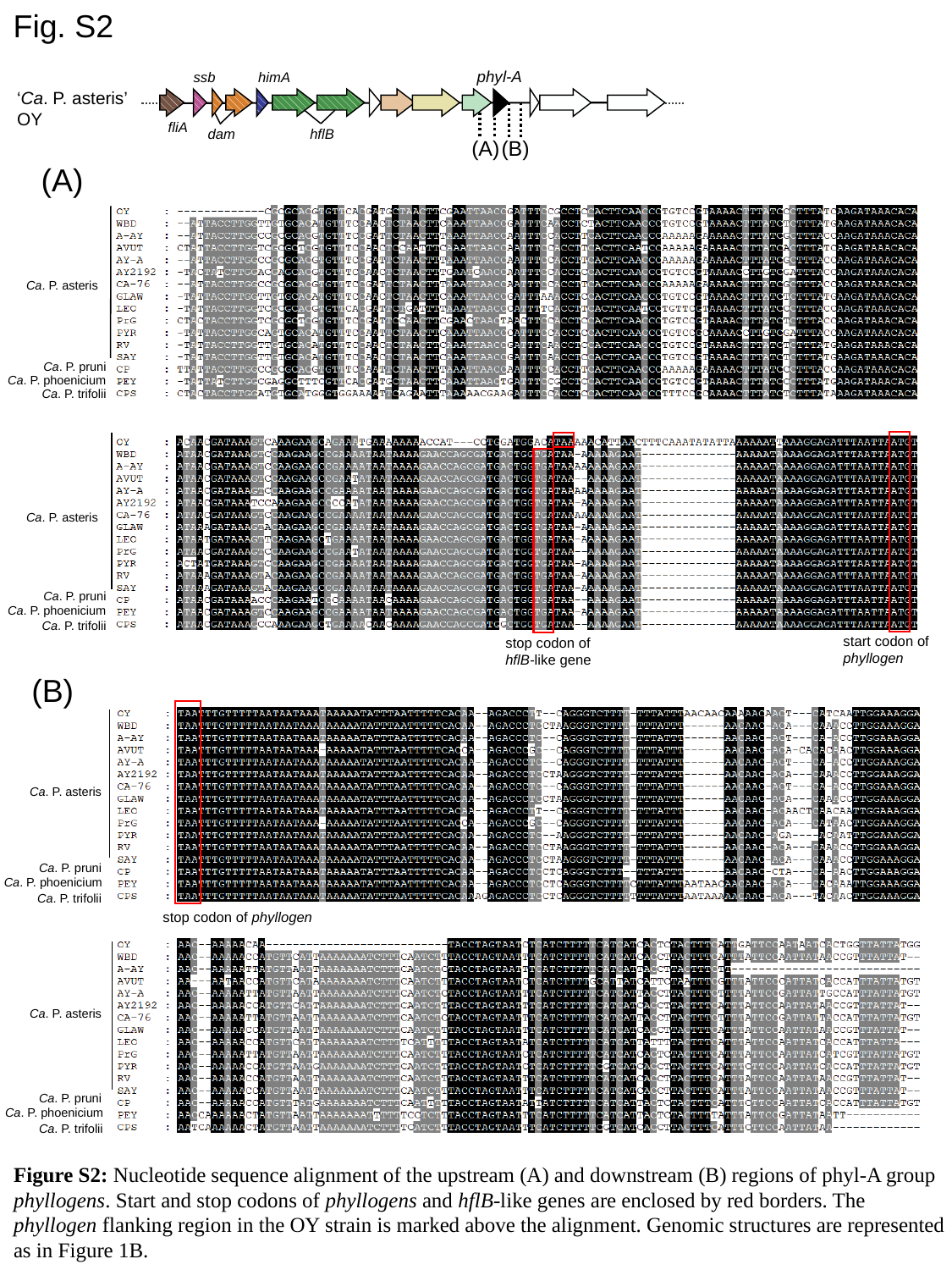

Fig. S2
phyl-A
ssb
himA
‘Ca. P. asteris’ OY
fliA
dam
hflB
(A)
(B)
(A)
Ca. P. asteris
Ca. P. pruni
Ca. P. phoenicium
Ca. P. trifolii
Ca. P. asteris
Ca. P. pruni
Ca. P. phoenicium
Ca. P. trifolii
start codon of phyllogen
stop codon of hflB-like gene
(B)
Ca. P. asteris
Ca. P. pruni
Ca. P. phoenicium
Ca. P. trifolii
stop codon of phyllogen
Ca. P. asteris
Ca. P. pruni
Ca. P. phoenicium
Ca. P. trifolii
Figure S2: Nucleotide sequence alignment of the upstream (A) and downstream (B) regions of phyl-A group phyllogens. Start and stop codons of phyllogens and hflB-like genes are enclosed by red borders. The phyllogen flanking region in the OY strain is marked above the alignment. Genomic structures are represented as in Figure 1B.

## Slide 3
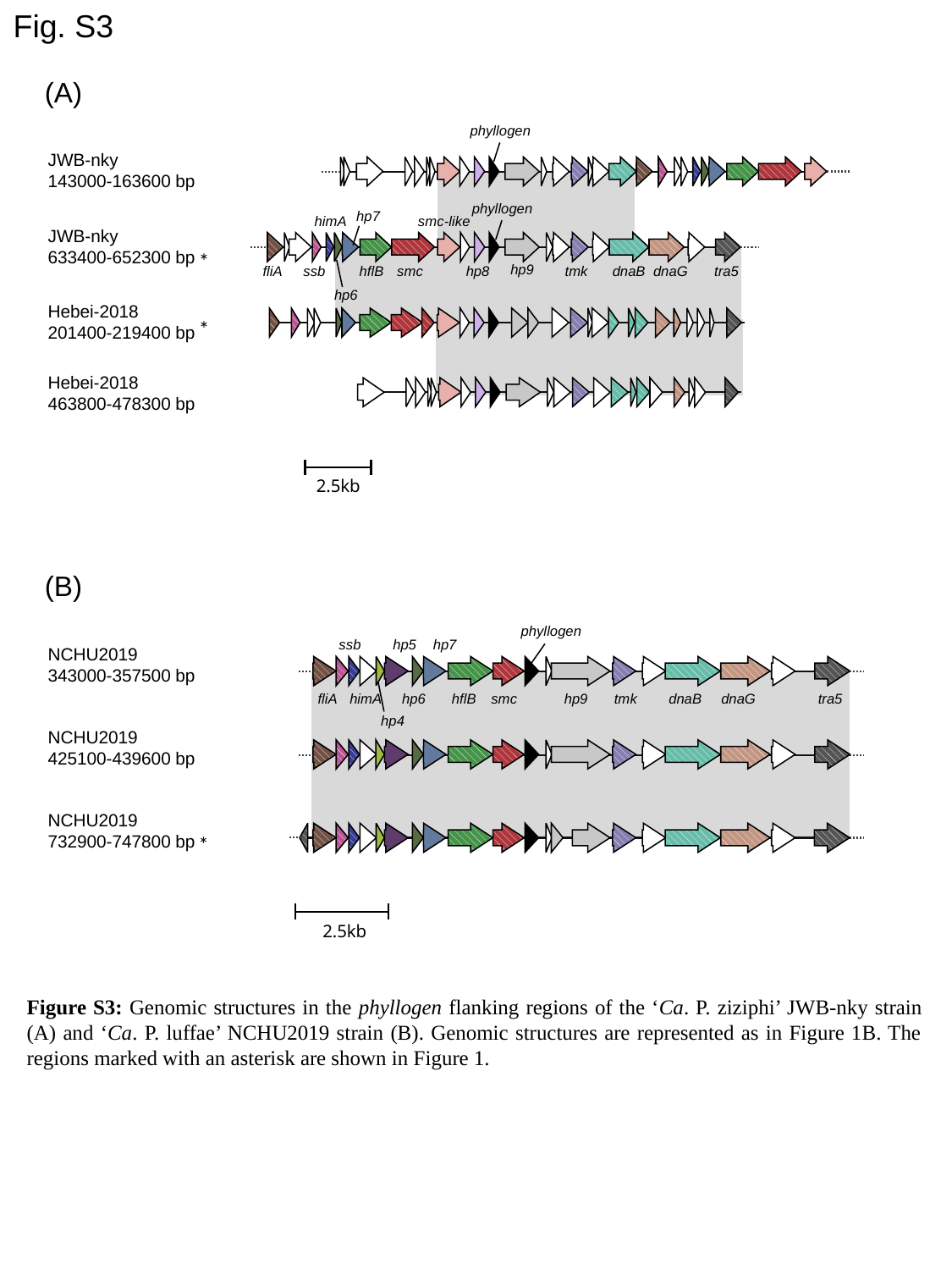

Fig. S3
(A)
phyllogen
JWB-nky
143000-163600 bp
phyllogen
hp7
himA
smc-like
JWB-nky
633400-652300 bp
*
hp9
fliA
ssb
hflB
smc
hp8
tmk
dnaB
dnaG
tra5
hp6
Hebei-2018
201400-219400 bp
*
Hebei-2018
463800-478300 bp
2.5kb
(B)
phyllogen
ssb
hp5
hp7
NCHU2019
343000-357500 bp
fliA
himA
hp6
hflB
smc
hp9
tmk
dnaB
dnaG
tra5
hp4
NCHU2019
425100-439600 bp
NCHU2019
732900-747800 bp
*
2.5kb
Figure S3: Genomic structures in the phyllogen flanking regions of the ‘Ca. P. ziziphi’ JWB-nky strain (A) and ‘Ca. P. luffae’ NCHU2019 strain (B). Genomic structures are represented as in Figure 1B. The regions marked with an asterisk are shown in Figure 1.

## Slide 4
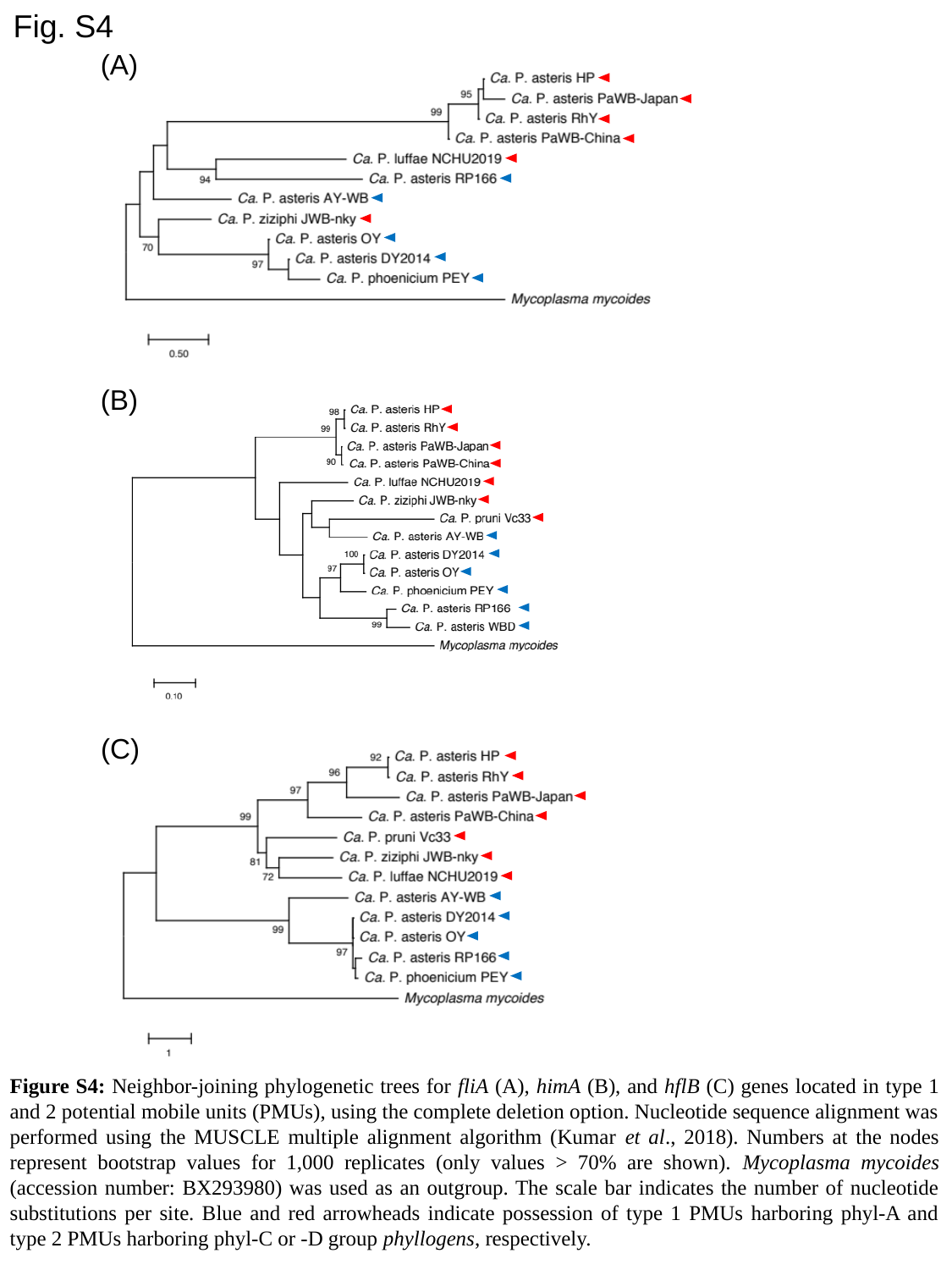

Fig. S4
(A)
(B)
(C)
Figure S4: Neighbor-joining phylogenetic trees for fliA (A), himA (B), and hflB (C) genes located in type 1 and 2 potential mobile units (PMUs), using the complete deletion option. Nucleotide sequence alignment was performed using the MUSCLE multiple alignment algorithm (Kumar et al., 2018). Numbers at the nodes represent bootstrap values for 1,000 replicates (only values > 70% are shown). Mycoplasma mycoides (accession number: BX293980) was used as an outgroup. The scale bar indicates the number of nucleotide substitutions per site. Blue and red arrowheads indicate possession of type 1 PMUs harboring phyl-A and type 2 PMUs harboring phyl-C or -D group phyllogens, respectively.

## Slide 5
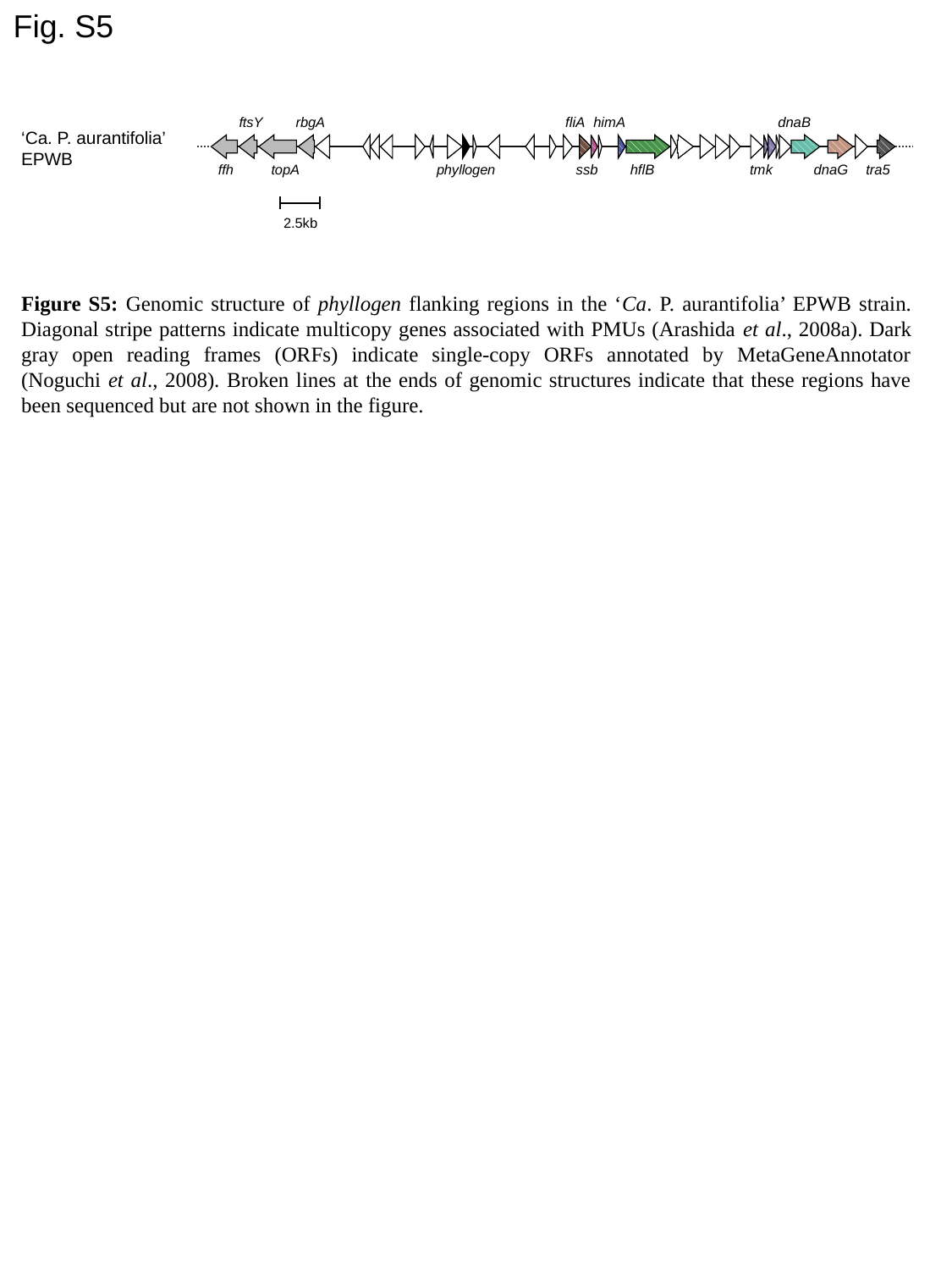

Fig. S5
ftsY
rbgA
fliA
himA
dnaB
‘Ca. P. aurantifolia’ EPWB
ffh
topA
phyllogen
ssb
hflB
tmk
dnaG
tra5
2.5kb
Figure S5: Genomic structure of phyllogen flanking regions in the ‘Ca. P. aurantifolia’ EPWB strain. Diagonal stripe patterns indicate multicopy genes associated with PMUs (Arashida et al., 2008a). Dark gray open reading frames (ORFs) indicate single-copy ORFs annotated by MetaGeneAnnotator (Noguchi et al., 2008). Broken lines at the ends of genomic structures indicate that these regions have been sequenced but are not shown in the figure.

## Slide 6
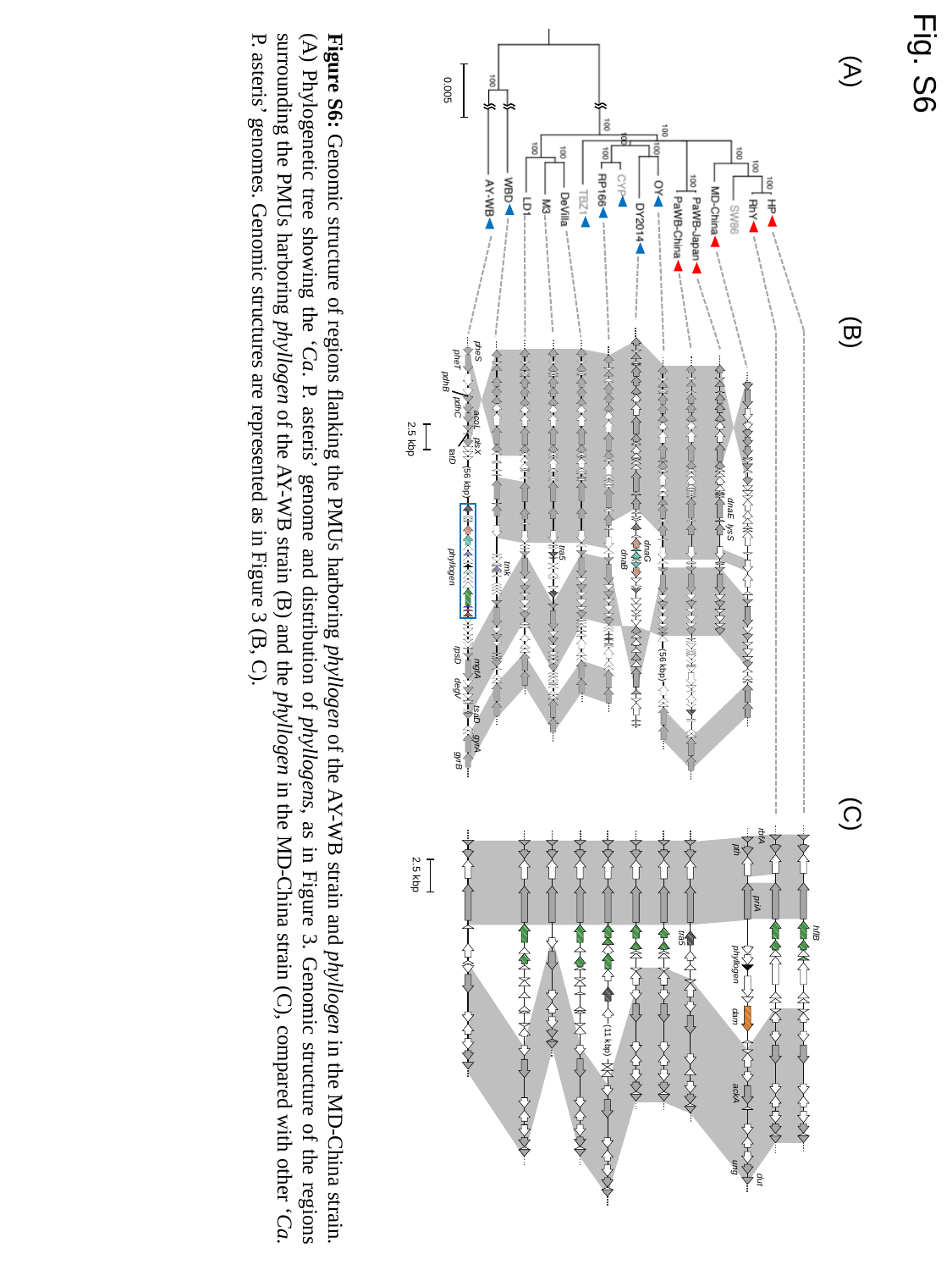

Fig. S6
(A)
0.005
(B)
dnaE
lysS
(56 kbp)
dnaG
dnaB
tra5
tmk
pheS
plsX
mgtA
gyrA
acoL
tsaD
(56 kbp)
gyrB
rpsD
degV
pdhC
pheT
tatD
phyllogen
pdhB
2.5 kbp
Figure S6: Genomic structure of regions flanking the PMUs harboring phyllogen of the AY-WB strain and phyllogen in the MD-China strain. (A) Phylogenetic tree showing the ‘Ca. P. asteris’ genome and distribution of phyllogens, as in Figure 3. Genomic structure of the regions surrounding the PMUs harboring phyllogen of the AY-WB strain (B) and the phyllogen in the MD-China strain (C), compared with other ‘Ca. P. asteris’ genomes. Genomic structures are represented as in Figure 3 (B, C).
(C)
rbfA
pth
2.5 kbp
priA
hflB
tra5
phyllogen
dam
(11 kbp)
ackA
ung
dut

## Slide 7
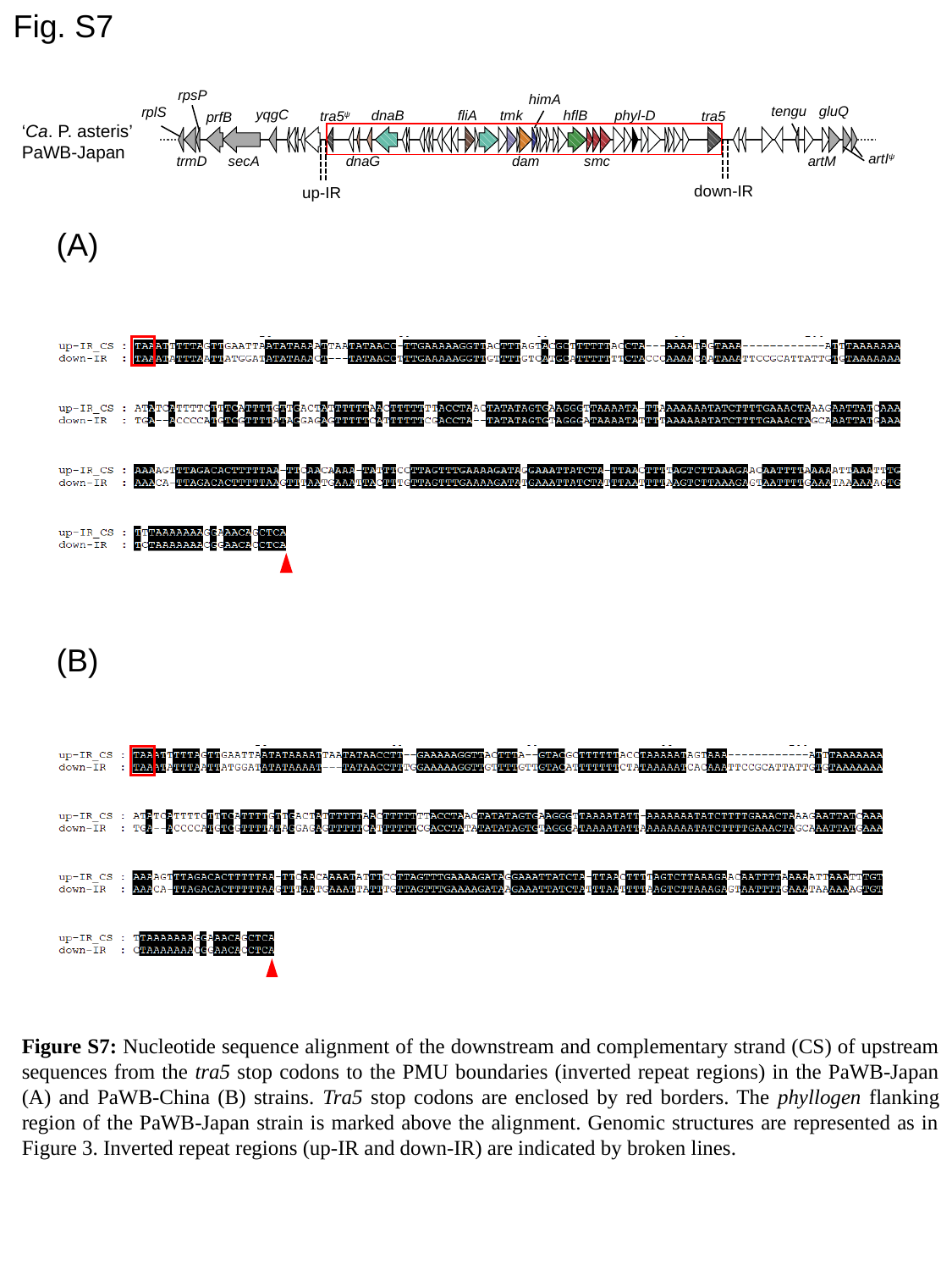

Fig. S7
rpsP
himA
tengu
gluQ
rplS
yqgC
phyl-D
dnaB
fliA
tmk
hflB
tra5
tra5ψ
prfB
‘Ca. P. asteris’ PaWB-Japan
artIψ
trmD
secA
dnaG
dam
smc
artM
down-IR
up-IR
(A)
v
(B)
v
Figure S7: Nucleotide sequence alignment of the downstream and complementary strand (CS) of upstream sequences from the tra5 stop codons to the PMU boundaries (inverted repeat regions) in the PaWB-Japan (A) and PaWB-China (B) strains. Tra5 stop codons are enclosed by red borders. The phyllogen flanking region of the PaWB-Japan strain is marked above the alignment. Genomic structures are represented as in Figure 3. Inverted repeat regions (up-IR and down-IR) are indicated by broken lines.
