# Supplementary material for: Potential mobile units drive the horizontal transfer of phytoplasma effector phyllogen genes
Source: Front Genet. 2023 May 11;14:1132432. doi: 10.3389/fgene.2023.1132432 (PMC10210161; doi:10.3389/fgene.2023.1132432)
Supplement: Supplementary file 3 [file DataSheet1.docx]

Supplementary Material

**Potential mobile units drive the horizontal transfer of phytoplasma effector *phyllogen* genes**

**Ryosuke Tokuda^1^, Nozomu Iwabuchi^1^, Yugo Kitazawa^1^, Takamichi Nijo^1^, Masato Suzuki^1^, Kensaku Maejima^1^, Kenro Oshima^2^, Shigetou Namba^1^, and Yasuyuki Yamaji^1*^**

^1^Department of Agricultural and Environmental Biology, Graduate School of Agricultural and Life Sciences, The University of Tokyo, Tokyo, Japan

^2^Faculty of Bioscience and Applied Chemistry, Hosei University, Tokyo, Japan

*** Correspondence:** Kensaku Maejima: amaejima@g.ecc.u-tokyo.ac.jp

**Supplementary Methods**

**Genome Sequencing**

phytoplasma DNA (HP, RhY, and PaWB strains) was enriched with DNA extracted from plants using the NEB Next Microbiome Enrichment kit (New England Biolabs, Ipswich, MA, USA), in accordance with the manufacturer’s instructions and as described previously (Nijo *et al*., 2021). For the RhY and PaWB-Japan strains, each genomic DNA library was constructed using the Illumina^®^ DNA Prep kit (M) Tagmentation (Illumina, Inc., San Diego, CA, USA), in accordance with the manufacturer’s instructions. Short-read sequencing was performed on a MiSeq instrument (Illumina) using the MiSeq reagent kit (version 3; 600 cycles) with a 2 × 150-bp paired-end read protocol. For the HP strain, the enriched DNA was subjected to genome amplification using the REPLI-g Mini kit (QIAGEN, Hilden, Germany) and size selection was performed using the short read eliminator XS kit (Circulomics, Baltimore, MD, USA). Long-read sequencing was performed on the PacBio Sequel II instrument (Pacific Biosciences, Menlo Park, CA, USA). The raw sequence data have been deposited in the DNA Data Bank of Japan (DDBJ) Sequence Read Archive (DRA: https://www. ddbj. nig. ac. jp/ dra/) under the accession numbers DRR423634 (RhY), DRR423635 (PaWB-Japan), and DRR423636 (HP), which are part of DRA study PRJDB14779. The raw reads from MiSeq were checked for quality using FastQC (ver. 0.11.8; http://www.bioinformatics.babraham.ac.uk/projects/fastqc/). The raw reads from PacBio were checked for quality using NanoPlot (Coster *et al*., 2018). Then, they were trimmed and selected using Filtlong (https://github.com/rrwick/Filtlong); the settings were as follows: min_length, 1000; keep_percent, 90; target_bases, 1000000000; and trim, split 500. The MiSeq reads, including those from HP strain sequenced previously (Nijo *et al*., 2021; accession number: DRR240862), were subjected to *de novo* metagenome assemblies using Unicycler (ver. 0.4.7; Wick *et al*., 2017). To improve the quality of the *de novo* assembly of the HP, RhY, and PaWB-Japan genomes, the assembled contigs were subjected to a BLASTn search against a nonredundant database for contigs derived from plants (> 10 kb). The raw reads from MiSeq and PacBio were mapped using Bowtie 2 (ver. 2.3.5.1; Langmead and Salzberg, 2012) and BWA-MEM (Li *et al*., 2010), respectively, and new libraries were constructed from the unmapped reads. For the RhY and PaWB-Japan strains, the new libraries were subjected to *de novo* metagenome assembly, as described above. For the HP strain, the new libraries from the MiSeq and PacBio reads were subjected to *de novo* metagenome hybrid assembly using Unicycler (ver. 0.4.7; Wick *et al*., 2017). The assembled contigs were subjected to BLASTn searching against a nonredundant database to identify putative phytoplasma contigs and then assigned to the draft genomes. The flanking sequences of the *phyllogen* containing contigs were determined using polymerase chain reaction, as described in the Materials and Methods section. The average depth of coverage was calculated by remapping all raw reads from MiSeq to the draft genomes and dividing the number of remapped reads by the total length of the draft genomes. The draft genomes were subjected to CheckM (Parks *et al*., 2015), with the genomes of ‘*Candidatus* Phytoplasma’ species used as a reference, to estimate genome completeness and contamination. Protein coding genes were annotated using the MetaGeneAnnotator (Noguchi *et al*., 2008) in DFAST (Tanizawa *et al*., 2018). rRNAs and tRNAs were annotated using Barrnap (https://github.com/tseemann/barrnap) and ARAGORN (Laslett and Canback, 2004), respectively, in DFAST (Tanizawa *et al*., 2018). All tools were run with default parameters. The draft genome sequences of the HP, RhY, and PaWB-Japan strains have been deposited in DDBJ under accession numbers BSDA00000000 (HP), BSCX00000000 (RhY), and BSCY00000000 (PaWB-Japan).

**Supplementary Results**

**Genome Sequencing of HP, RhY, and PaWB-Japan strains**

The draft genomes of HP, RhY, and PaWB-Japan strains were sequenced to identify their *phyllogen* flanking sequences. The enriched DNA samples from plants infected with these strains respectively were subjected to Illumina sequencing analysis in RhY and PaWB-Japans and PacBio sequencing analysis in HP strains. A total of 3,138,422 and 1,930,602 paired-end reads ranging from 35–151 bp were obtained in RhY and PaWB-Japan strains. A total of 247,274 reads that included 2,490,994,292 bp were obtained in HP strains. The raw reads of HP strain were filtered to 65,663 reads that included 1,000,004,953 bp. These reads were used to de novo assemblies and 34, 45, and 104 contigs were obtained in HP, RhY, and PaWB-Japan strains. From these contigs, we detected full-length *phyllogen* sequences and *phyllogen* flanking regions whose lengths were 60,522, 17,516, and 26,779 bp, respectively. Those regions of RhY and PaWB-Japan strains were prolonged to 51,917 and 63,736 bp using PCR. Combing these sequences and assembled contigs, the draft genomes were constructed. Contig numbers decreased to 43 and 103 in RhY and PaWB-Japan strains. Total lengths were 641,311, 609,849, and 776,015 bp in HP, RhY, and PaWB-Japan genomes (Fig. S1; Table S5; 68–135% those of the complete genomes of ‘*Ca*. P. asteris’-related strains). These N50 contig lengths were 140,653 in HP, 41,738 in RhY, and 45,936 in PaWB-Japan strains (Table S5). The completeness values of these genomes were all 99% and the contamination values were all below 1% (Table S5). These results suggested that most regions of these genomes could be sequenced.

**References**

Coster, W. D., D’Hert, S., Schultz, D. T., Cruts, M., Broeckhoven, C. V. (2018). NanoPack: visualizing and processing long read sequencing data. *Bioinformatics.* 34, 2666–2669. doi: 10.1093/bioinformatics/bty149

Langmead, B., Salzberg, S. L. (2012). Fast gapped-read alignment with Bowtie 2. *Nat. Methods.* 9, 357–359. doi: 10.1038/nmeth.1923

Laslett, D., Canback, B. (2004). ARAGORN, a program to detect tRNA genes and tmRNA genes in nucleotide sequences. *Nucleic. Acids. Res.* 32, 11–16. doi: 10.1093/nar/gkh152

Li, H., Durbin, R. (2010). Fast and accurate long-read alignment with Burrows-Wheeler transform. *Bioinformatics.* 26, 589–595. doi: 10.1093/bioinformatics/btp698

Nijo, T., Iwabuchi, N., Tokuda, R., Suzuki, T., Matsumoto, O., Miyazaki, A., et al. (2021). Enrichment of phytoplasma genome DNA through a methyl-CpG binding domain-mediated method for efficient genome sequencing. *J. Gen. Plant. Pathol.* 87, 154–163. doi: 10.1007/s10327-021-00993-z

Noguchi, H., Taniguchi, T., Itoh, T. (2008). MetaGeneAnnotator: detecting species-specific patterns of ribosomal binding site for precise gene prediction in anonymous prokaryotic and phage genomes. *DNA Res.* 15, 387–396. doi: 10.1093/dnares/dsn027

Parks, D. H., Imelfort, M., Skennerton, C. T., Hugenholtz, P., Tyson, G. W. (2015). CheckM: assessing the quality of microbial genomes recovered from isolates, single cells, and metagenomes. *Genome Res.* 25, 1043–1055. doi: 10.1101/gr.186072.114

Tanizawa, Y., Fujisawa, T., Nakamura, Y. (2018). DFAST: a flexible prokaryotic genome annotation pipeline for faster genome publication. *Bioinformatics.* 34, 1037–1039. doi: 10.1093/bioinformatics/btx713

Wick, R. R., Judd, L. M., Gorrie, C. L., Holt, K. E. (2017). Unicycler: resolving bacterial genome assemblies from short and long sequencing reads. *PLoS Comput. Biol.* 13, e1005595. doi: 10.1371/journal.pcbi.1005595
